# Author Correction: Improved ethanol electrooxidation performance by shortening Pd–Ni active site distance in Pd–Ni–P nanocatalysts

**DOI:** 10.1038/s41467-020-15019-z

**Published:** 2020-03-09

**Authors:** Lin Chen, Lilin Lu, Hengli Zhu, Yueguang Chen, Yu Huang, Yadong Li, Leyu Wang

**Affiliations:** 1 State Key Laboratory of Chemical Resource Engineering, Beijing University of Chemical Technology, Beijing, 100029 China; 2 School of Chemistry and Chemical Engineering, Wuhan University of Science and Technology, Wuhan, 430081 China; 3 Department of Chemistry, Tsinghua University, Beijing, 100086 China; 4 Department of Materials Science & Engineering, University of California Los Angeles, California, 90095 USA

Correction to: *Nature Communications* 10.1038/ncomms14136, published online 10 January 2017.

This Article contains an error in Figs. 4c and 4e, which were placed in wrong order by error.

The correct version of Fig. 4 is:



The error has not been corrected in the PDF or HTML versions of the Article.

