# Supplementary material for: Improved ethanol electrooxidation performance by shortening Pd–Ni active site distance in Pd–Ni–P nanocatalysts
Source: Nat Commun. 2017 Jan 10;8:14136. doi: 10.1038/ncomms14136 (PMC5234093; doi:10.1038/ncomms14136)
Supplement: Supplementary Information — Supplementary Figures, Supplementary Tables, Supplementary Method and Supplementary References [file ncomms14136-s1.pdf]

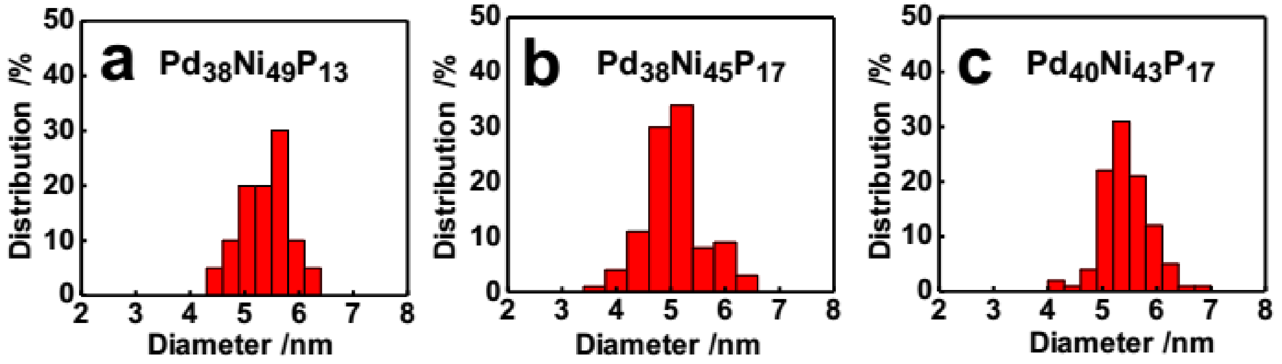

Supplementary Figure 1. Size distribution of these Pd-Ni-P ternary NPs. The size distribution profiles of $Pd_{38}Ni_{49}P_{13}$ (a), $Pd_{38}Ni_{45}P_{17}$ (b) and $Pd_{40}Ni_{43}P_{17}$ (c), respectively. The average sizes of these NPs are 5.5 ± 1.0 nm, 5.3 ± 1.0 nm, 5.3 ± 0.5 nm, respectively.

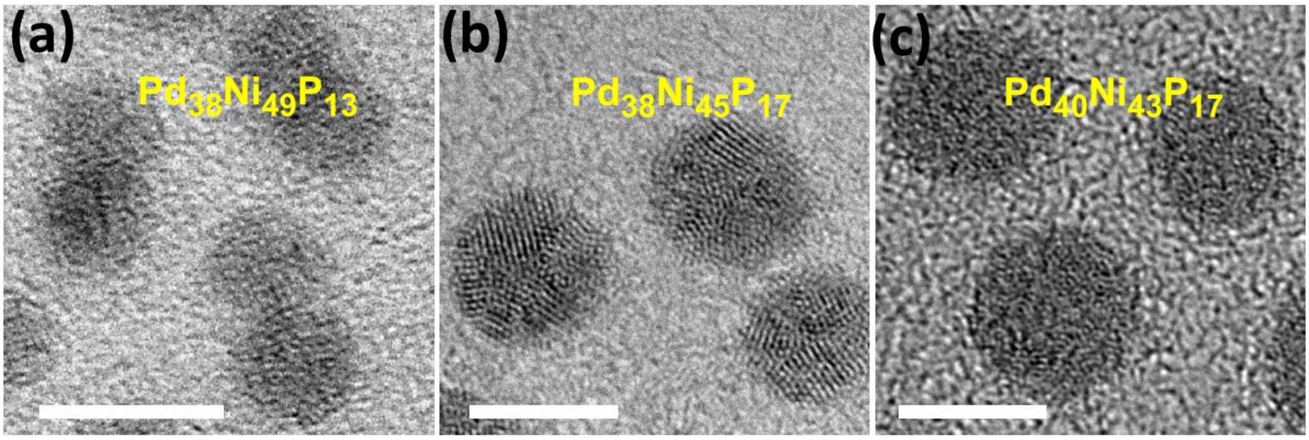

Supplementary Figure 2. HRTEM images. High resolution transmission electron microscope (HRTEM) images of (a) $Pd_{38}Ni_{49}P_{13}$, (b) $Pd_{38}Ni_{45}P_{17}$ and (c) $Pd_{40}Ni_{43}P_{17}$ nanoparticles prepared with different phosphorization time. (a): 5 min (260 °C); (b): 1 h (260 °C); (c): 1 h (260 °C) + 1 h (290 °C). Scale bar in **a-c**, 5 nm.

As shown in Supplementary Figure 2a, the $Pd_{38}Ni_{49}P_{13}$ NPs are Pd/Ni-P heterodimers and then transfer into spherical nanoparticles ($Pd_{38}Ni_{45}P_{17}$) with large Pd domains in Ni-P matrixes after phosphorization for 1 h at 260 °C (Supplementary Figure 2b). And then the crystalline Pd domains in Supplementary Figure 2b were disappeared after further phosphorization at 290 °C for another 1 h (Supplementary Figure 2c). The composition of these as-prepared NPs was determined *via* the inductively coupled plasma mass spectrometry (ICP-MS).

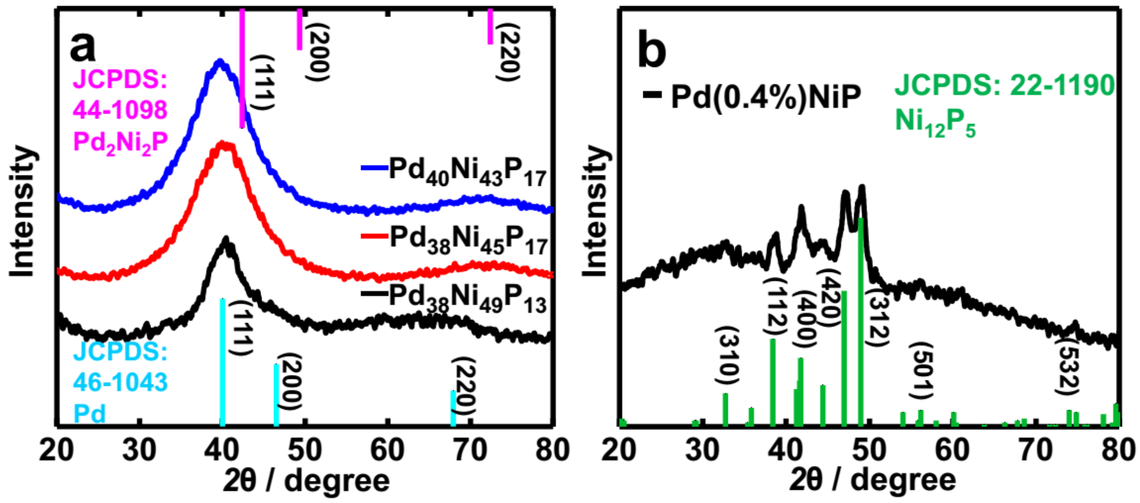

Supplementary Figure 3. XRD patterns. X-ray diffraction (XRD) patterns of the Pd-Ni-P ternary NPs (a) and $Ni_{12}P_5$ NPs with only 0.4% of Pd-doping (b).

To probe the spatial distribution of Pd, Ni and P species in the $Pd_{38}Ni_{49}P_{13}$ heterodimers (Supplementary Figure 4), $Pd_{38}Ni_{45}P_{17}$ (Supplementary Figure 5) and $Pd_{40}Ni_{43}P_{17}$ NPs (Supplementary Figure 6), HAADF-STEM images as well as elemental mapping were performed. The Pd, Ni and P signals unambiguously illustrates the existence of these three elements in every nanoparticle. From the line-scan results, the dissolution of Ni is clearly observed after further phosphorization, which is confirmed by the chemical composition analysis of ICP-MS. Due to the resolution limit of instruments, we cannot get the elemental image of single heterodimer. However, in combination with the HRTEM image, the heterostructure of the heterodimer is still clear from Supplementary Figure 4b, where the palladium (Pd) and nickel (Ni) are shown as large domains. There is obvious phase segregation in the composite maps of Pd and Ni (Supplementary Figure 4b) as well as Pd and P (Supplementary Figure 4d). Meanwhile, as shown in Supplementary Figure 4c, the Ni and P species are homogeneously distributed, suggesting that the phosphor (P) element mainly combines with Ni rather than Pd and the Pd species are presented as big domains.

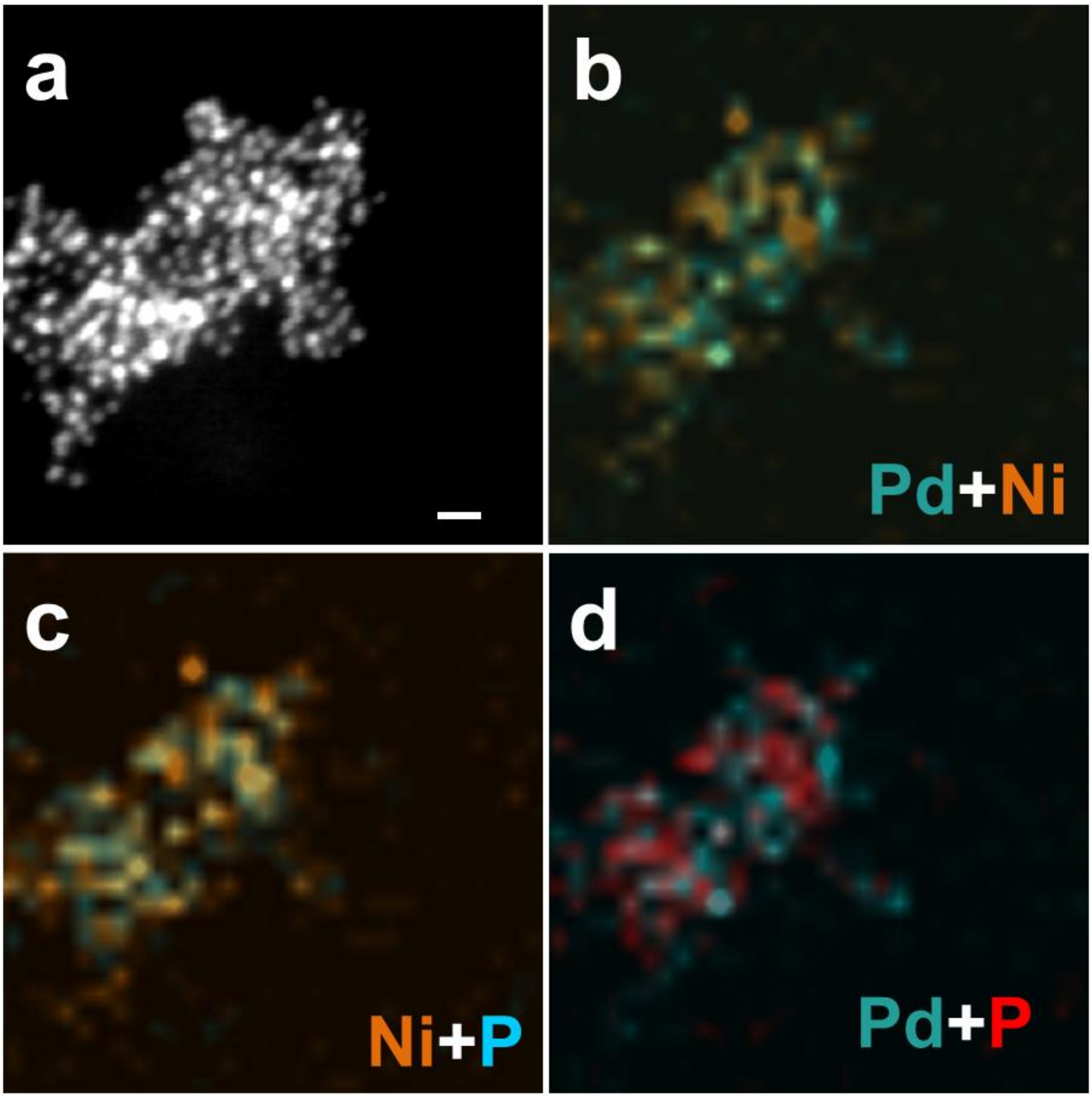

Supplementary Figure 4. STEM image and elemental imaging. HAADF-STEM image and the corresponding elemental mapping analysis of $Pd_{38}Ni_{49}P_{13}$ heterodimers. The element ratio of NPs was detected *via* ICP-MS. It is noteworthy that, to make the image clear, the color of phosphor (P) in Supplementary Figure 4c is changed into blue from red shown in Supplementary Figure 4d. Scale bar in **a**, 10 nm.

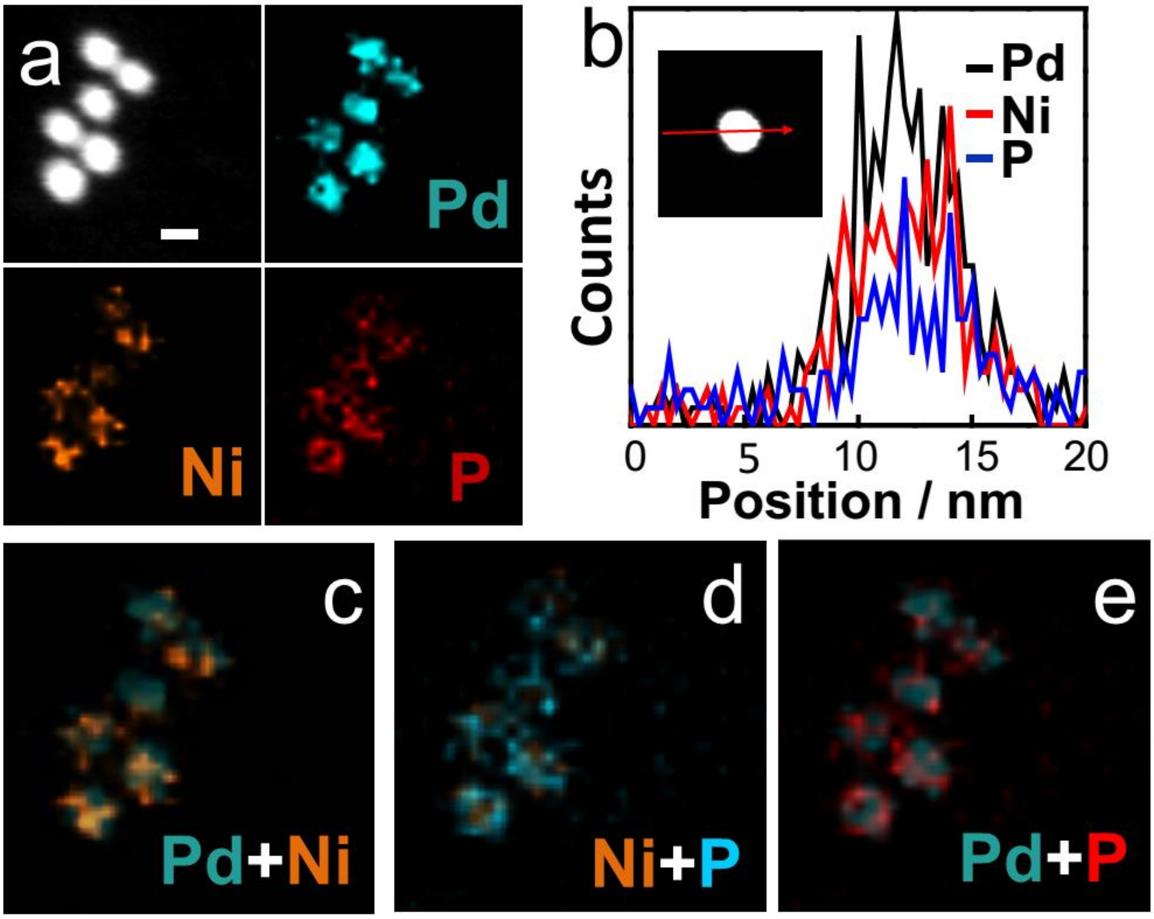

Supplementary Figure 5. STEM image and elemental imaging. HAADF-STEM image and the corresponding elemental mapping analysis (a) and line scan (b) of $Pd_{38}Ni_{45}P_{17}$ NPs. The element ratio of NPs was detected *via* ICP-MS. Scale bar in **a**, 5 nm.

According to the elemental mapping results (Supplementary Figure 5b), obvious phase segregation is observed in the composite map of Pd and Ni, indicating that the Pd-dispersion in Ni-P matrixes is still very low even though the phosphorization time is prolonged to 1 h (260 °C). However, after phosphorization at 290 °C for another 1 h, the Pd is highly dispersed into the Ni-P matrix in the $Pd_{40}Ni_{43}P_{17}$ ternary NPs. As shown in Supplementary Figure 6b, the distribution areas of Ni and Pd are similar to the sizes of the nanoparticles and no phase segregation can be observed, but the Pd is dominantly presented as Pd (0) rather than Pd-P (Supplementary Figure 6d), which is in line with the XPS results.

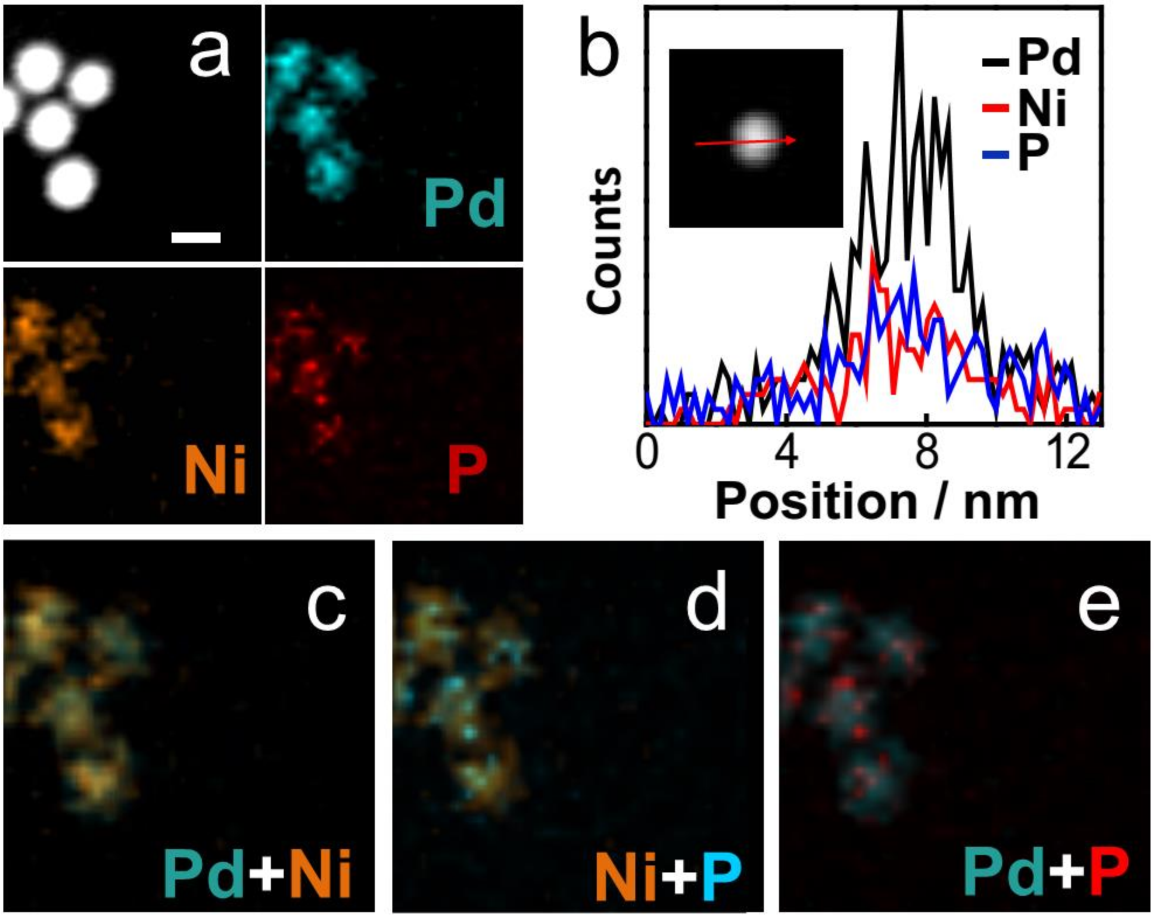

Supplementary Figure 6. STEM image and elemental imaging. HAADF-STEM image and the corresponding elemental mapping analysis (a) and line scan (b) of $Pd_{40}Ni_{43}P_{17}$ NPs. The element ratio of NPs was detected *via* ICP-MS. Scale bar in **a**, 5 nm.

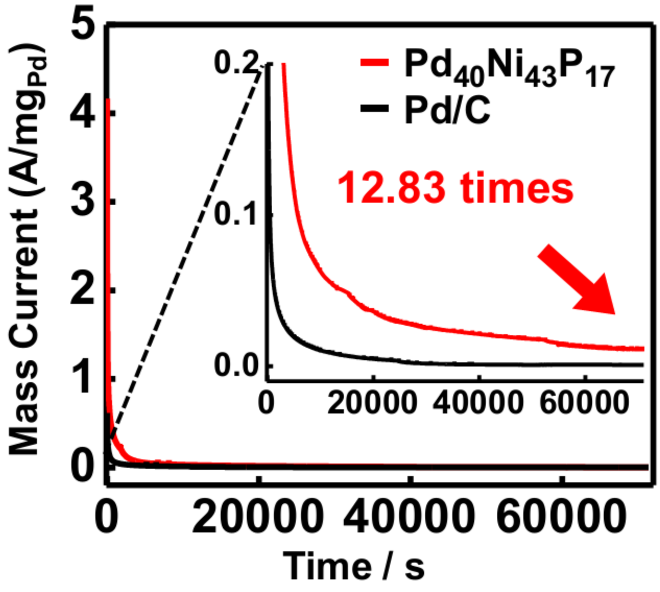

Supplementary Figure 7. Electrocatalytic durability tests. Long-term stability tests of these NPs and commercial Pd/C catalysts. Chronoamperometry curves measured in 1.0 M NaOH + 1.0 M C_2H_5OH (the corresponding potential was held at 0.8 V during the measurements).

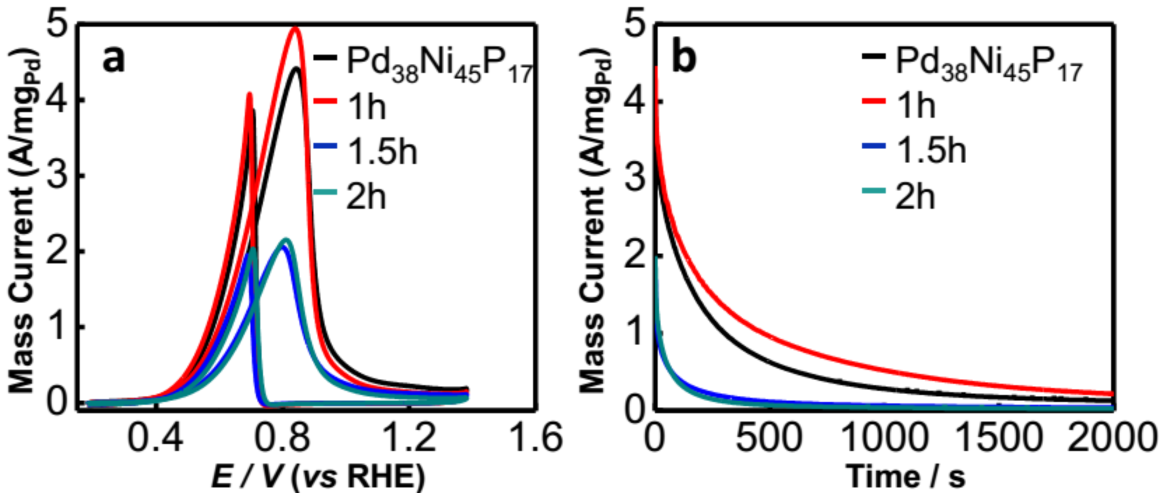

Supplementary Figure 8. Electrocatalytic performance tests. CVs (a) and chronoamperometry curves (b) of $Pd_{38}Ni_{45}P_{17}$ NPs with different phosphorization time in 1.0 M NaOH and 1.0 M C_2H_5OH at 100 mVs^{-1} (the corresponding potential was held at 0.8V during the measurements).

As mentioned in Supplementary Table 1, the Ni would dissolve during the phosphorization, which would influence the activity and durability of the nanocatalysts. As shown in Supplementary Figure 8, if the $Pd_{38}Ni_{45}P_{17}$ NPs were treated with phosphorization (290 °C) for 1 h to get the $Pd_{40}Ni_{43}P_{17}$ NPs, these as-obtained $Pd_{40}Ni_{43}P_{17}$ NPs demonstrated the best electrocatalysis activity and durability. Further prolonging the phosphorization time would suffer from bad activity and stability. This can be attributed to the heavy loss of Ni, which may greatly decrease the dissociative adsorption of H_2O (forming OH radicals on Ni surface) and subsequent oxidative removal of carbonaceous poison such as CO on active Pd sites. As a result, the optimization of phosphorization time is necessary toward good activity and durability.

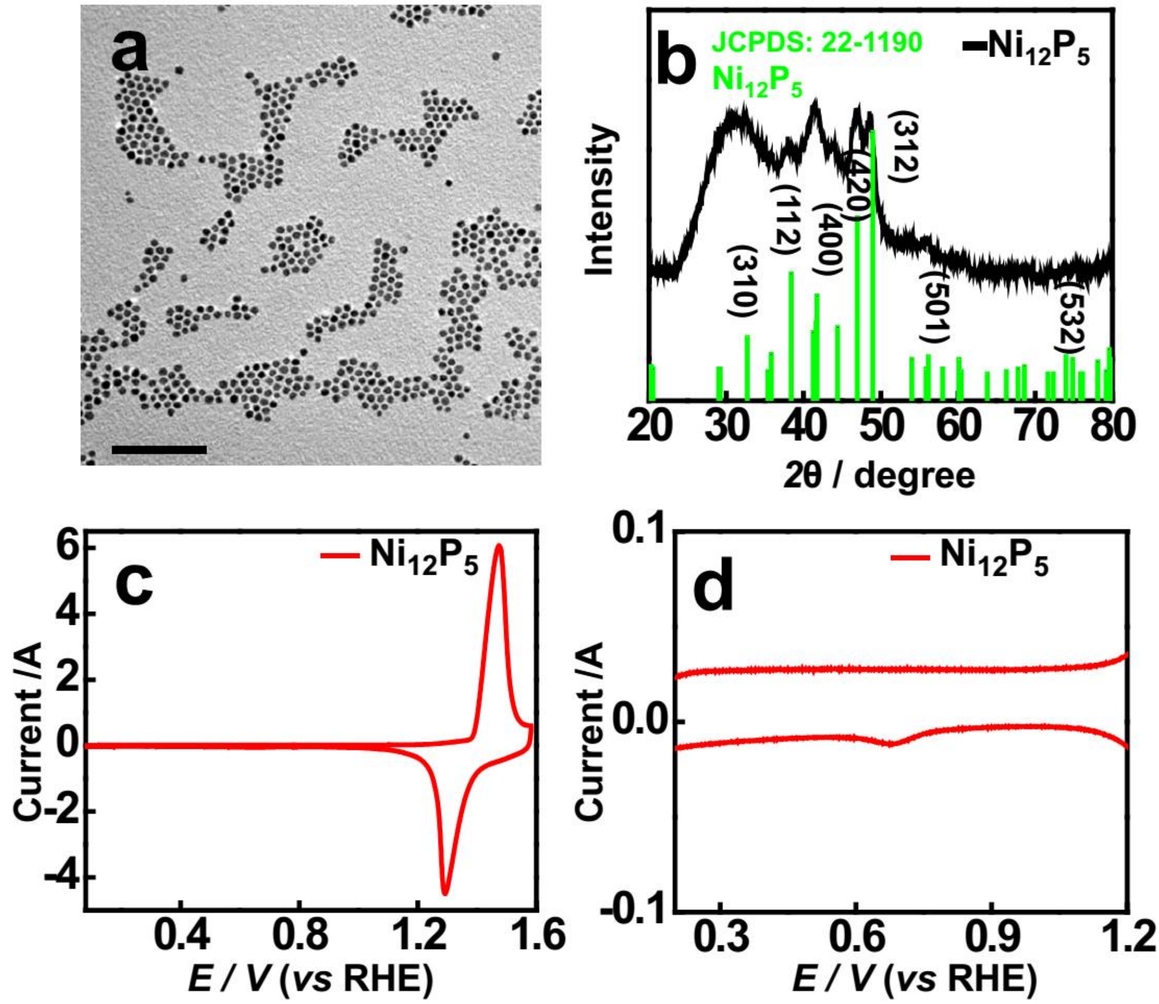

Supplementary Figure 9. TEM, XRD and catalytic activity measurements of $Ni_{12}P_5$ nanocrystals. (a) TEM image and (b) XRD pattern of the as-prepared $Ni_{12}P_5$ nanocrystals; CVs of $Ni_{12}P_5$ nanocrystals in (c) 1 M NaOH and (d) 1 M NaOH + 1.0 M C_2H_5OH, respectively. Scale bar in **a**, 100 nm.

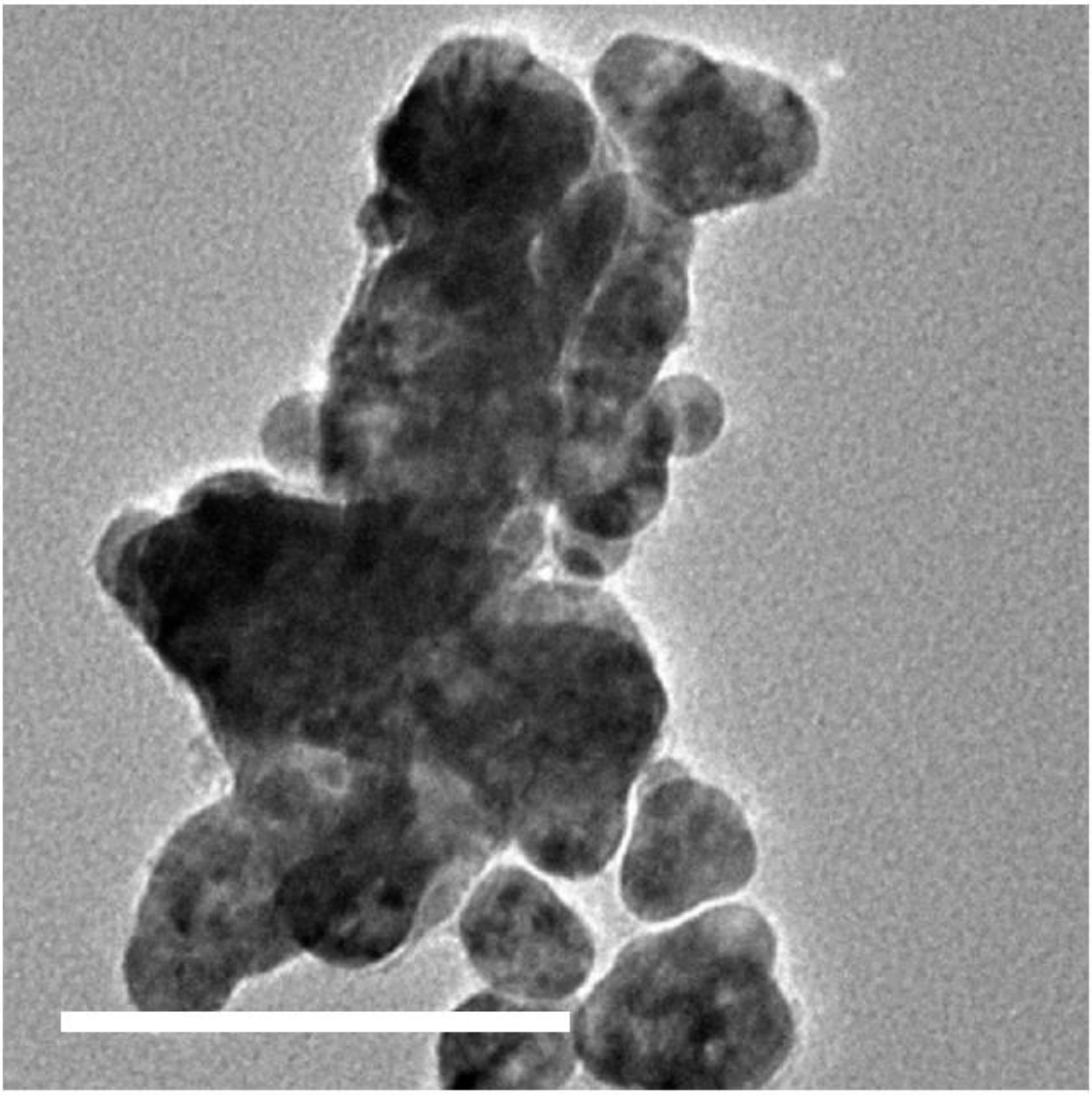

Supplementary Figure 10. TEM image of Pd-Ni aggregates prepared in the absence of TOP. The synthesis procedures followed the same protocol for preparation of Pd-Ni-P NPs, only without adding TOP reagent. Scale bar, 100 nm.

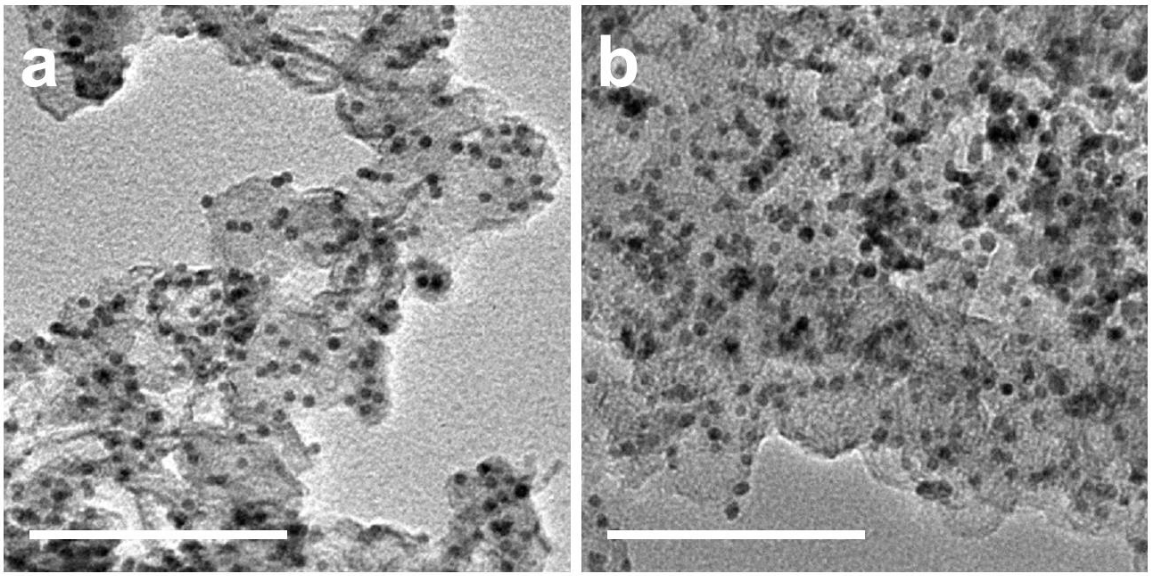

Supplementary Figure 11. TEM images of nanocatalysts before and after EOR. TEM images of $Pd_{40}Ni_{43}P_{17}$ NPs attached to carbon before (a) and after (b) EOR. Scale bar in **a**-**b**, 100 nm.

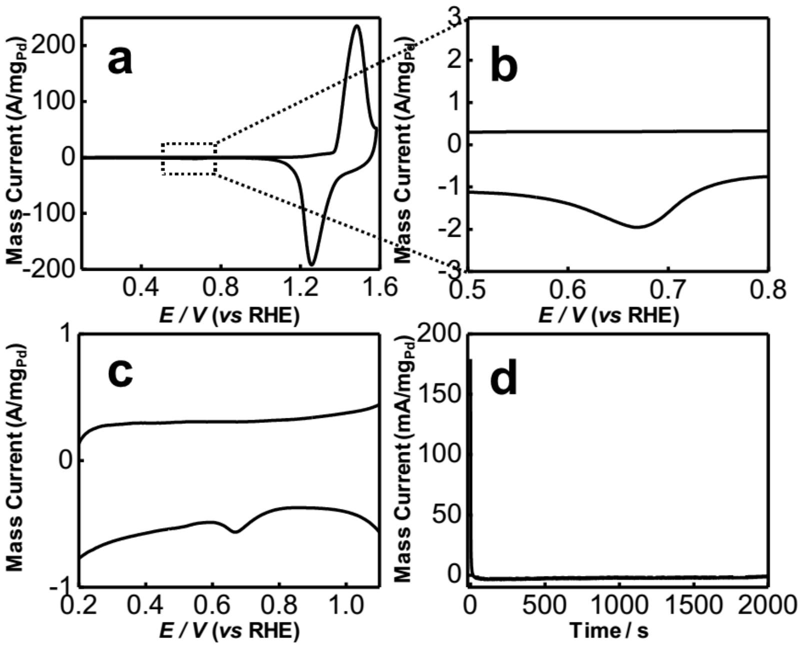

Supplementary Figure 12. Electrocatalytic property evaluation. a, b) CVs obtained on Pd-Ni-P NPs (Pd/Ni = 0.4%, atomic ratio) in 1.0 M NaOH solution at a scan rate of 100 mVs^{-1}; c) CVs in 1.0 M NaOH and 1.0 M C_2H_5OH at 100 mVs^{-1}; d) Chronoamperometry curve measured in 1.0 M NaOH and 1.0 M C_2H_5OH (the corresponding potential was held at 0.8 V during the measurements).

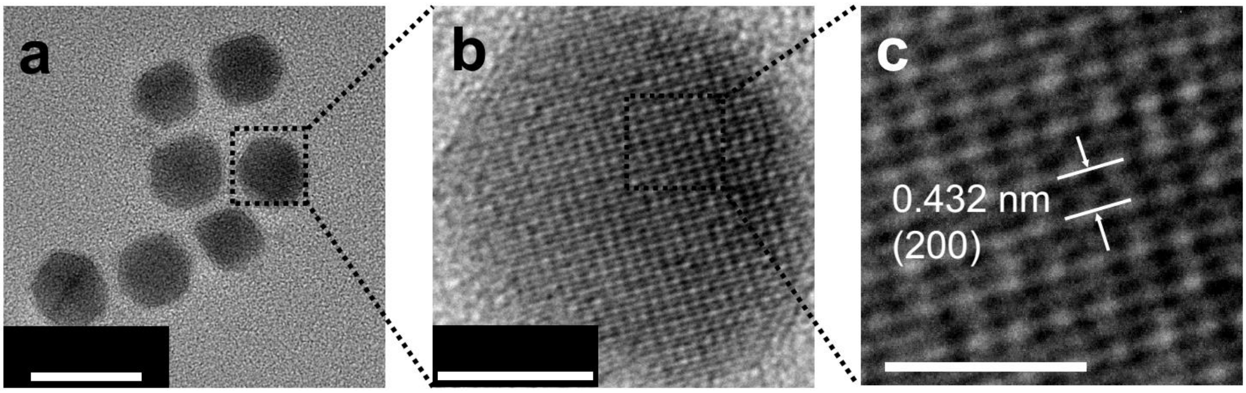

Supplementary Figure 13. HRTEM images. HRTEM images of the Pd-Ni-P NPs with atomic ratio of 0.4% (Pd/Ni). According to the HRTEM and XRD (Supplementary Figure 3b) results, these NPs can be indexed to $Ni_{12}P_5$ (JCPDS: 22-1190). Scale bar in **a** (20 nm), in **b** (5 nm) and in **c** (2 nm).

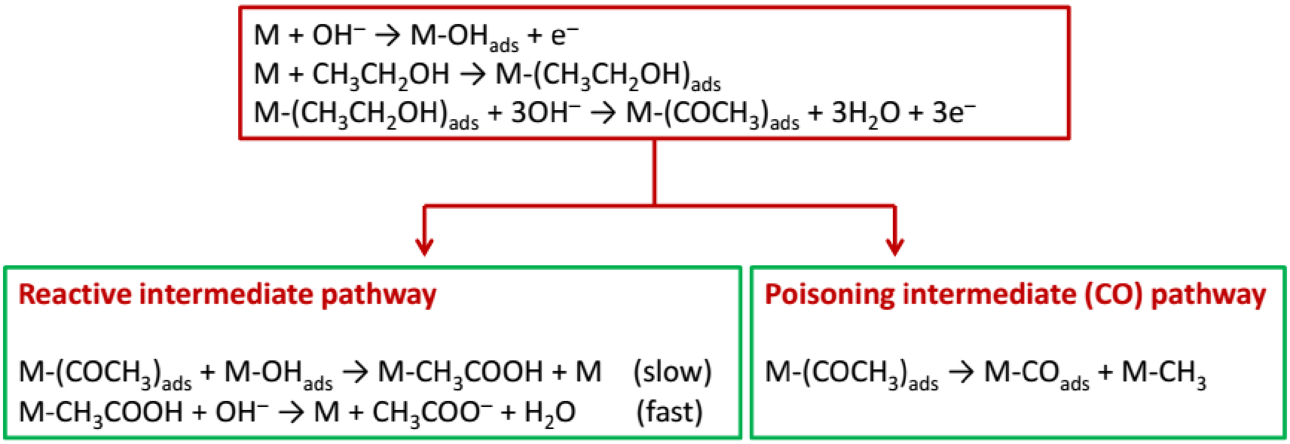

Supplementary Figure 14. Scheme for the reaction pathway. The reactive-intermediate and/or the poisoning-intermediate (CO) pathway for ethanol electrooxidation reaction in alkaline media.

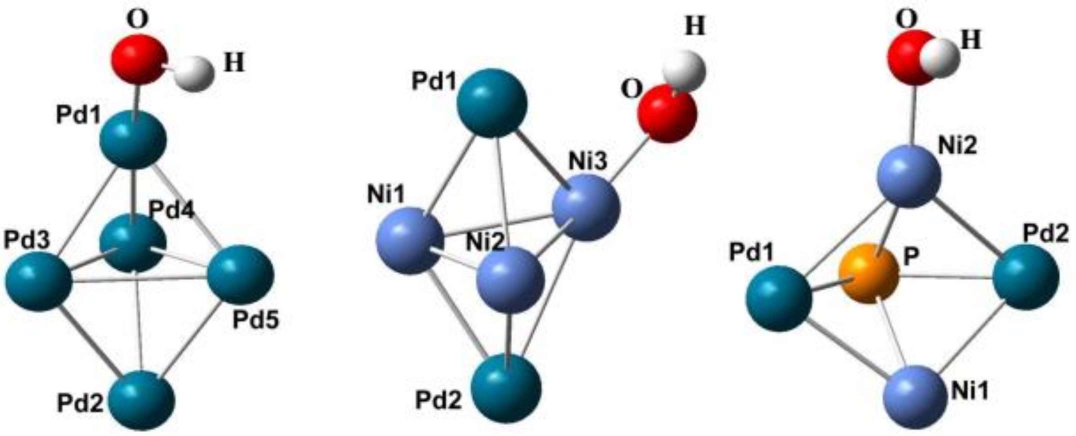

Supplementary Figure 15. Optimal geometries for DFT calculation. The optimized geometries of OH^{-} adsorption complex on Pd_5, Ni_3Pd_2 and PNi_2Pd_2 cluster.

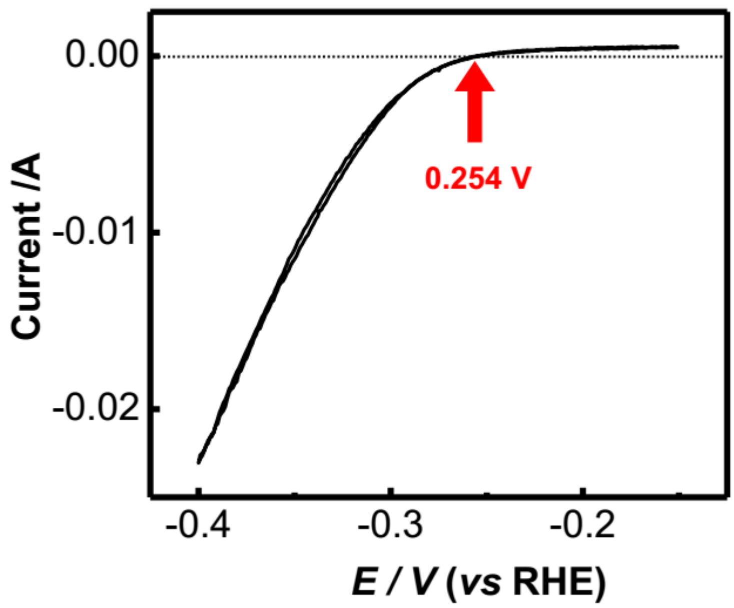

Supplementary Figure 16. Electrode calibration. CVs result of RHE calibration in 0.5 M H_2SO_4 solution.

Supplementary Table 1. Influence of phosphorization time on the Pd/Ni ratio in the Pd-Ni-P ternary NPs.

Sample	Phosphorization time/ h		Pd/Ni (mole ratio)
S1	0	($Pd_{38}Ni_{45}P_{17}$)	0.84
S2	1	($Pd_{40}Ni_{43}P_{17}$)	0.93
S3	1.5		1.22
S4	2		1.38

Note please, here the influence of phosphorization time (290 °C) on the Pd/Ni atomic ratio in the ternary NPs was investigated using S1 ($Pd_{38}Ni_{45}P_{17}$) as precursors.

All the element contents in the nanoparticles were carried out *via* ICP-MS. As shown in Supplementary Table 1, with the prolonging of phosphorization time, the Pd-to-Ni ratio was increased step by step, suggesting the dissolution of Ni during the phosphorization.

Supplementary Table 2. Comparison of the activity and durability of various Pd-containing nanomaterials for EOR in alkaline media.

Catalyst	electrolyte	Pd loading (μg/cm^2)	Mass activity (A/mg$_{Pd}$)	Improvement compared to Pd/C	Durability	Ref.
**$Pd_{40}Ni_{43}P_{17}$ NP/C electrocatalyst**	**1M NaOH 1M ethanol**	**22.57**	**4.945**	**6.88 times**	**215.4 mA/mg$_{Pd}$ after 2000 s**	***In this work***
PdCo nanotube arrays on carbon cloth	1M KOH 1M ethanol	23.4	1.5	4.0 times	175 mA/mg$_{Pd}$ after 550 s	[1]*Angew. Chem. Int. Ed.* ***2015*** *, 54 , 3669*
Pd_7/Ru_1 bimetallic nanodendrites	1M KOH 1M NaOH	56.62	1.15	3.0 times	14 mA/mg$_{Pd}$ after 3600 s	[2]*Nanoscale.* ***2015****, 7, 12445*
Pd supported on B,N-codoped graphene	1M NaOH 1M ethanol	28	2.156	2.5 times	206 mA/mg$_{Pd}$ after 3600 s	[3]*J. Mater. Chem. A.* ***2016****, 4 (13), 4929*
Pd-PEDOT/graphene nanocomposites	1M KOH 1M ethanol	86	0.417	2.78 times	39.2 mA/mg$_{Pd}$ after 4000 s	[4]*J. Mater. Chem. A.* ***2015****, 3 (3), 1077*
flower-like ordered Pd_3Pb nanocrystals	0.5M NaOH 1M ethanol	—	0.51	1.41 times	30 mA/mg$_{Pd}$ after 1000 s	[5]*J. Power Sources* ***2016****, 301, 160*
$Pd_{83}Ni_{17}$ HNS aerogel electrocatalyst	1M NaOH 1M ethanol	20	3.63	5.6 times	75 mA/mg$_{Pd}$ after 2000 s	[6]*Angew. Chem. Int. Ed.* ***2015****, 54, 1310*
PdPt nanowires	0.5M NaOH 1M ethanol	71.4	0.94	1.9 times	39 mA/mg$_{Pd}$ after 200 s	[7]*Adv. Mater.* ***2012****, 24 (17), 2326*
Pd-$NiCoO_x$/C catalyst	0.1M KOH 0.5M ethanol	—	0.43	2.05 times	55 mA/mg$_{Pd}$ after 3000 s	[8]*J. Power Sources.* ***2015****, 273, 631*

Supplementary Table 3. Synthetic conditions for nanocatalyst precursors.

Product	$Pd(acac)_2/Ni(acac)_2$ (mmol)	OAm/TOP (mL)	Temperature (°C)	Reaction time
$Pd_{47}Ni_{36}P_{17}$	0.6/0.4	10/1.5	260	1 h
$Pd_{38}Ni_{49}P_{13}$	0.5/0.5	10/1.5	260	5 min
$Pd_{38}Ni_{45}P_{17}$	0.5/0.5	10/1.5	260	1 h
$Pd_{31}Ni_{53}P_{16}$	0.4/0.6	10/1.5	260	1 h

Supplementary Table 4. Phosphorization conditions of NPs.

Final product	Nanoparticle precursors (Pd/Ni) 0.2 mmol	OAm/TOP (mL)	Temperature (°C)	Reaction time
$Pd_{54}Ni_{30}P_{16}$	$Pd_{47}Ni_{36}P_{17}$ (6/4)	10/1	290	1 h
$Pd_{40}Ni_{43}P_{17}$	$Pd_{38}Ni_{45}P_{17}$ (5/5)	10/1	290	1 h
$Pd_{32}Ni_{50}P_{18}$	$Pd_{31}Ni_{53}P_{16}$ (4/6)	10/1	290	1 h

Supplementary Table 5. Hirshfeld atomic charge distribution, OH^- absorption energy, and *OH radical desorption energy.

		Pd_5 cluster	Ni_3Pd_2 cluster	PNi_2Pd_2 cluster
atomic charge (*a. u.*)	Pd1	0.0036	-0.0368	-0.0161
	Pd2	0.0036	-0.0368	-0.0161
	Pd3	-0.0025	—	—
	Pd4	0.0012	—	—
	Pd5	-0.0025	—	—
	Ni1	—	0.0172	0.0645
	Ni2	—	0.0293	0.0667
	Ni3		0.0293	-0.0978
	P	—	—	-0.0978
adsorption energy (OH^-, kcal/mol)		103.4,	112.0	115.7
desorption energy (*OH radical, kcal/mol)		59.6,	126.5	44.2

Supplementary Method 1

Density functional theory (DFT) calculations to understand the function of incorporating Ni and P in the Pd-Ni-P ternary nanocatalysts.

Experiments: As shown in Supplementary Figure 15, three cluster models (PNi_2Pd_2, Ni_3Pd_2 and Pd_5) were constructed to study the formation of *OH on the investigated nanocatalysts. The density functional theory (DFT) calculations are performed using the Gaussian 09 suite of programs.[9] Geometries of all chemical systems are fully optimized without symmetry constraints by employing Becke's three-parameter hybrid functional B3LYP,[10, 11] the 6-311G (d, p) basis set was used for hydrogen, oxygen, and phosphorus atoms, and the LAN2DZ basis set for palladium atoms with effective core pseudo potential. For open-shell species the unrestricted formulation is used to optimize the ground state geometries. All optimized structures are confirmed to be real minima by frequency calculation at the same level of theory (no imaginary frequency), and unscaled zero-point energies (ZPE) are abstracted from frequency analysis to make thermochemical correction to electronic energies. The preference of adsorption site of hydroxide ion (OH^-) on catalysts and adsorption energies were discussed based on the ZPE-corrected electronic energies. Hirshfeld charge analysis was performed to investigate the atomic charge distribution of the investigated catalyst cluster models.

Detailed discussions: We first constructed cluster models of the nanocatalysts and performed the relatively accurate hybrid density functional theory (B3LYP) calculations to understand how the nature of the catalyst affects the EOR, in other words, to investigate the formation and dissociation of *OH on the nanocatalysts. The stablest geometries of adsorption complex of OH^- on cluster models (PNi_2Pd_2, Ni_3Pd_2 and Pd_5) for the calculation are shown in Supplementary Figure 14. In alkaline medium the formation and dissociation of *OH is consist of three separated step, i.e. adsorption of hydroxide ion on catalyst, electron loss controlled by applied external electric field, and desorption of *OH from catalysts. The chemical properties of catalysts would show significant effects on the first and third step because both of them are chemical processes, but little effects on the second step

because of its electrochemical nature. As shown in Supplementary Figure 15, OH^- shows preferential adsorption on Ni atom for Ni_3Pd_2 and PNi_2Pd_2 clusters, which is in good agreement with experimental observation.[12] This can be ascribed to the electrostatic attraction between OH^- and Ni atom which carries significant positive charge demonstrated by Hirshfeld charge analysis. However, the P atom carries significant negative charge and Pd atom is almost electrically neutral in Pd-Ni-P ternary catalysts (Supplementary Table 5). This is also confirmed by our XPS experimental observation. Our preliminary calculation data show that the *OH is mainly formed on the surface of Ni.[12] The adsorption energies on Pd_5, Ni_3Pd_2 and PNi_2Pd_2 are calculated to be 103.4, 112.0 and 115.7 kcal/mol (Supplementary Table 5), respectively, indicating that binary and ternary catalysts are more favorable for chemical absorption of OH^-, and the dopant of P atom further improves the adsorption energy of OH^- by 3.7 kcal/mol. After electron loss the adsorbed OH radical is formed, and the desorption energies of OH radical from Pd_5, Ni_3Pd_2 and PNi_2Pd_2 are calculated to be 59.6, 126.5 and 44.2 kcal/mol, respectively. The lowest desorption energy of OH radical for PNi_2Pd_2 catalysts indicates that P doping improves the production of free OH radicals, thus facilitates the formation of CH_3COOH (Figure 8). Indeed, the incorporation of Ni and P into Pd can facilitate the formation of OH_{ads} on the nanostructure surface owing to the exposed oxophilic Ni sites and the changes in electronic structure,[12-14] which has been confirmed by the XPS measurements. Therefore, the incorporation of Ni and P in the ternary nanocatalysts drives the EOR preferentially through the efficient reactive-intermediate pathway.

Supplementary References

1. Wang AL, He XJ, Lu XF, Xu H, Tong YX, Li GR. Palladium-cobalt nanotube arrays supported on carbon fiber cloth as high-performance flexible electrocatalysts for ethanol oxidation. *Angew. Chem.-Int. Edit.* **54**, 3669-3673 (2015).
2. Zhang K, Bin D, Yang BB, Wang CQ, Ren FF, Du YK. Ru-assisted synthesis of Pd/Ru nanodendrites with high activity for ethanol electrooxidation. *Nanoscale* **7**, 12445-12451 (2015).
3. Liu Q, Fan JC, Min YL, Wu T, Lin Y, Xu QJ. B, N-codoped graphene nanoribbons supported Pd nanoparticles for ethanol electrooxidation enhancement. *J. Mater. Chem. A* **4**, 4929-4933 (2016).
4. Yue RR*, et al.* Facile one-pot synthesis of Pd-PEDOT/graphene nanocomposites with hierarchical structure and high electrocatalytic performance for ethanol oxidation. *J. Mater. Chem. A* **3**, 1077-1088 (2015).
5. Jana R, Subbarao U, Peter SC. Ultrafast synthesis of flower-like ordered Pd3Pb nanocrystals with superior electrocatalytic activities towards oxidation of formic acid and ethanol. *J. Power Sources* **301**, 160-169 (2016).
6. Cai B, Wen D, Liu W, Herrmann AK, Benad A, Eychmuller A. Function-led design of aerogels: self-assembly of alloyed PdNi hollow nanospheres for efficient electrocatalysis. *Angew. Chem.-Int. Edit.* **54**, 13101-13105 (2015).
7. Zhu CZ, Guo SJ, Dong SJ. PdM (M = Pt, Au) Bimetallic Alloy Nanowires with Enhanced Electrocatalytic Activity for Electro-oxidation of Small Molecules. *Adv. Mater.* **24**, 2326-2331 (2012).
8. Wang W, Yang Y, Liu YQ, Zhang Z, Dong WK, Lei ZQ. Hybrid NiCoOx adjacent to Pd nanoparticles as a synergistic electrocatalyst for ethanol oxidation. *J. Power Sources* **273**, 631-637 (2015).
9. Frisch MJ. Gaussian09, revision B.01, Gaussian, Inc., Wallingford, CT. *Gaussian09, revision B.01, Gaussian, Inc., Wallingford, CT*, (2009).
10. Becke AD. Density functional thermochemistry. Ⅲ. The role of exact exchange. *J. Chem. Phy.* **114**, 3958-3967 (1993).
11. C. Lee W, Yang RGP. Development of the Colle-Salvetti correlation-energy for mulain to a functional of the electron density. *Phy. Rev. B* **37**, 785-789 (1988).
12. Wang Y, Shi FF, Yang YY, Cai WB. Carbon supported Pd-Ni-P nanoalloy as an efficient catalyst for ethanol electro-oxidation in alkaline media. *J. Power Sources* **243**, 369-373 (2013).
13. Ham DJ*, et al.* Palladium-nickel alloys loaded on tungsten carbide as platinum-free anode electrocatalysts for polymer electrolyte membrane fuel cells. *Chem. Commun.* **47**, 5792-5794 (2011).
14. Zhao M, Abe K, Yamaura S, Yamamoto Y, Asao N. Fabrication of Pd-Ni-P metallic glass nanoparticles and their application as highly durable catalysts in methanol electro-oxidation. *Chem. Mater.* **26**, 1056-1061 (2014).
